# Supplementary material for: The global viralization of policies to contain the spreading of the COVID-19 pandemic: Analyses of school closures and first reported cases
Source: PLoS One. 2021 Apr 1;16(4):e0248828. doi: 10.1371/journal.pone.0248828 (PMC8016240; doi:10.1371/journal.pone.0248828)
Supplement: S1 File — (DOCX) [file pone.0248828.s001.docx]

**S1 File. Dates and sources**

|  | **Country** | **First case** | **Close schools** | **Source 1** | **Source 2** |
| --- | --- | --- | --- | --- | --- |
| 1 | Afghanistan | 24-02-2020 | 14-03-2020 | <https://en.wikipedia.org/wiki/2020_coronavirus_pandemic_in_Afghanistan> | <https://www.aljazeera.com/news/2020/03/war-ravaged-afghanistan-battles-threat-coronavirus-200316135224827.html> |
| 2 | Albania | 08-03-2020 | 09-03-2020 | <https://www.albaniandailynews.com/index.php?idm=40177&mod=2> | <https://en.wikipedia.org/wiki/2020_coronavirus_pandemic_in_Albania> |
| 3 | Algeria | 25-02-2020 | 12-03-2020 | <https://en.unesco.org/themes/education-emergencies/coronavirus-school-closures> | <https://en.wikipedia.org/wiki/2020_coronavirus_pandemic_in_Algeria> |
| 4 | Angola | 19-03-2020 | No | <https://www.nytimes.com/reuters/2020/03/21/world/africa/21reuters-health-coronavirus-angola.html> | |
| 5 | Argentina | 03-03-2020 | 15-03-2020 |  | <https://www.clarin.com/politica/coronavirus-argentina-alberto-fernandez-encabeza-reunion-definir-suspenden-clases-medidas-toman_0_fknnQf-s.html> |
| 6 | Armenia | 01-03-2020 | 02-03-2020 | <https://www.azatutyun.am/a/30485453.html> | <https://en.wikipedia.org/wiki/2020_coronavirus_pandemic_in_Armenia> |
| 7 | Australia | 25-01-2020 | No |  | <https://en.wikipedia.org/wiki/2020_coronavirus_pandemic_in_Australia> |
| 8 | Austria | 25-02-2020 | 11-03-2020 | <https://www.usnews.com/news/world/articles/2020-03-11/austria-closing-schools-over-coronavirus-as-border-checks-take-effect> | <https://en.wikipedia.org/wiki/2020_coronavirus_pandemic_in_Austria> |
| 9 | Azerbaijan | 28-02-2020 | 02-03-2020 | <https://en.wikipedia.org/wiki/2020_coronavirus_pandemic_in_Azerbaijan> | <https://www.civilnet.am/news/2020/03/02/Coronavirus-Armenian-Government-Introduces-School-Closures-Establishes-Quarantine-Center-in-Tsaghkadzor/377468> |
| 10 | Bahamas | 15-03-2020 | No | <https://en.wikipedia.org/wiki/2020_coronavirus_pandemic_in_the_Bahamas> | |
| 11 | Bahrain | 21-02-2020 | 26-02-2020 | <https://english.alarabiya.net/en/News/gulf/2020/02/25/Bahrain-suspends-all-private-public-schools-amid-coronavirus-outbreak.html> | <https://en.wikipedia.org/wiki/2020_coronavirus_pandemic_in_Bahrain> |
| 12 | Bangladesh | 07-03-2020 | 16-03-2020 | <https://en.wikipedia.org/wiki/2020_coronavirus_pandemic_in_Bangladesh> | <https://www.thedailystar.net/bangladesh-all-educational-institutions-closed-till-march-31-1881541> |
| 13 | Barbados | 17-03-2020 | No | <https://en.wikipedia.org/wiki/2020_coronavirus_pandemic_in_Barbados> | |
| 14 | Belarus | 28-02-2020 | No | <https://en.wikipedia.org/wiki/2020_coronavirus_pandemic_in_Belarus> | <https://belsat.eu/en/news/no-official-quarantine-planned-for-belarus/> |
| 15 | Belgium | 04-02-2020 | 13-03-2020 | <https://www.aa.com.tr/en/europe/belgium-portugal-to-shut-schools-over-coronavirus/1764338> | <https://en.wikipedia.org/wiki/2020_coronavirus_pandemic_in_Belgium> |
| 16 | Belize | No |  |  |  |
| 17 | Benin | 16-03-2020 | No | <https://en.wikipedia.org/wiki/2020_coronavirus_pandemic_in_Africa> | |
| 18 | Bhutan | 06-03-2020 | 10-03-2020 | <https://en.wikipedia.org/wiki/2020_coronavirus_pandemic_in_Bhutan> | <https://en.unesco.org/themes/education-emergencies/coronavirus-school-closures> |
| 19 | Bolivia (Plurinational State of) | 10-03-2020 | 12-03-2020 | <https://en.unesco.org/themes/education-emergencies/coronavirus-school-closures> | <https://en.wikipedia.org/wiki/2020_coronavirus_pandemic_in_South_America> |
| 20 | Bosnia and Herzegovina | 05-03-2020 | 11-03-2020 | <https://en.unesco.org/themes/education-emergencies/coronavirus-school-closures> | <https://en.wikipedia.org/wiki/2020_coronavirus_pandemic_in_Bosnia_and_Herzegovina> |
| 21 | Botswana | No | 20-03-2020 | <https://en.unesco.org/themes/education-emergencies/coronavirus-school-closures> | <https://en.wikipedia.org/wiki/2020_coronavirus_pandemic_in_Africa#Botswana> |
| 22 | Brazil | 26-02-2020 | 12-03-2020 | <https://en.unesco.org/themes/education-emergencies/coronavirus-school-closures> | <https://en.wikipedia.org/wiki/2020_coronavirus_pandemic_in_South_America> |
| 23 | Brunei Darussalam | 09-03-2020 | No | <https://en.wikipedia.org/wiki/2020_coronavirus_pandemic_in_Brunei> | |
| 24 | Bulgaria | 08-03-2020 | 16-03-2020 | <https://en.unesco.org/themes/education-emergencies/coronavirus-school-closures> | <https://en.wikipedia.org/wiki/2020_coronavirus_pandemic_in_Bulgaria> |
| 25 | Burkina Faso | 09-03-2020 | 16-03-2020 | <https://en.unesco.org/themes/education-emergencies/coronavirus-school-closures> | |
| 26 | Burundi | No |  | <https://www.theeastafrican.co.ke/scienceandhealth/Africa-coronavirus-cases-to-rise-as-some-undetected/3073694-5498212-o7o1n3z/index.html> | |
| 27 | Cote d'Ivoire | 11-03-2020 | 17-03-2020 | <https://en.wikipedia.org/wiki/2020_coronavirus_pandemic_in_Africa> | |
| 28 | Cabo Verde | 16-03-2020 |  | <https://www.africanews.com/2020/03/20/coronavirus-hub-impact-of-outbreak-across-africa/> | |
| 29 | Cambodia | 27-01-2020 | 16-03-2020 | <https://en.unesco.org/themes/education-emergencies/coronavirus-school-closures> | <https://en.wikipedia.org/wiki/2020_coronavirus_pandemic_in_Cambodia> |
| 30 | Cameroon | 06-03-2020 | 18-03-2020 | <https://en.wikipedia.org/wiki/2020_coronavirus_pandemic_in_Africa> | |
| 31 | Canada | 25-01-2020 | 16-03-2020 | <https://montrealgazette.com/news/local-news/a-timeline-of-the-novel-coronavirus-in-quebec> | |
| 32 | Central African Republic | 14-03-2020 |  | <https://en.wikipedia.org/wiki/2020_coronavirus_pandemic_in_Africa> | |
| 33 | Chad | 19-03-2020 | 20-03-2020 | <https://en.wikipedia.org/wiki/2020_coronavirus_pandemic_in_Africa> | |
| 34 | Chile | 03-03-2020 | 16-03-2020 | <https://en.unesco.org/themes/education-emergencies/coronavirus-school-closures> | <https://en.wikipedia.org/wiki/2020_coronavirus_pandemic_in_South_America> |
| 35 | China |  |  |  |  |
| 36 | Colombia | 06-03-2020 | 16-03-2020 | <https://en.unesco.org/themes/education-emergencies/coronavirus-school-closures> | <https://en.wikipedia.org/wiki/2020_coronavirus_pandemic_in_South_America> |
| 37 | Comoros | No reported cases |  |  |  |
| 38 | Congo | 14-03-2020 | No | <https://en.wikipedia.org/wiki/2020_coronavirus_pandemic_in_Africa> | |
| 39 | Cook Islands | |  |  |  |
| 40 | Costa Rica | 06-03-2020 | 17-03-2020 | <https://en.unesco.org/themes/education-emergencies/coronavirus-school-closures> | <https://en.wikipedia.org/wiki/2020_coronavirus_pandemic_in_Costa_Rica> |
| 41 | Croatia | 25-02-2020 | 12-03-2020 | <https://en.unesco.org/themes/education-emergencies/coronavirus-school-closures> | <https://en.wikipedia.org/wiki/2020_coronavirus_outbreak_in_Croatia> |
| 42 | Cuba | 11-03-2020 | No | <https://en.wikipedia.org/wiki/2020_coronavirus_pandemic_in_Cuba> | <https://www.diariolasamericas.com/america-latina/cuba-no-cierra-fronteras-prohibe-nasobucos-y-niega-crisis-n4195297> |
| 43 | Cyprus | 09-03-2020 | 13-03-2020 | <https://en.unesco.org/themes/education-emergencies/coronavirus-school-closures> | <https://en.wikipedia.org/wiki/2020_coronavirus_pandemic_in_Cyprus> |
| 44 | Czech Republic | 01-03-2020 | 10-03-2020 | <https://en.wikipedia.org/wiki/2020_coronavirus_pandemic_in_the_Czech_Republic> | |
| 45 | Democratic People's Republic of Korea | | | |  |
| 46 | Democratic Republic of the Congo | 14-03-2020 | No | <https://en.wikipedia.org/wiki/2020_coronavirus_pandemic_in_Africa> | |
| 47 | Denmark | 27-02-2020 | 16-03-2020 | <https://www.statista.com/statistics/1102237/coronavirus-cases-development-in-denmark/> | <https://en.unesco.org/themes/education-emergencies/coronavirus-school-closures> |
| 48 | Djibouti | 18-03-2020 | No | <https://en.wikipedia.org/wiki/2020_coronavirus_pandemic_in_Africa> | |
| 49 | Dominica |  |  |  |  |
| 50 | Dominican Republic | 01-03-2020 | 17-03-2020 | <https://en.wikipedia.org/wiki/2020_coronavirus_pandemic_in_the_Dominican_Republic> | <https://www.expansion.com/empresas/transporte/2020/03/18/5e7185f7e5fdea9e608b4654.html> |
| 51 | Ecuador | 29-02-2020 | 13-03-2020 | <https://en.unesco.org/themes/education-emergencies/coronavirus-school-closures> | https://en.wikipedia.org/wiki/2020_coronavirus_pandemic_in_South_America |
| 52 | Egypt | 14-02-2020 | 16-03-2020 | <https://en.wikipedia.org/wiki/2020_coronavirus_pandemic_in_Africa> | <https://en.unesco.org/themes/education-emergencies/coronavirus-school-closures> |
| 53 | El Salvador | 18-03-2020 | 11-03-2020 | <https://en.unesco.org/themes/education-emergencies/coronavirus-school-closures> | <https://www.usnews.com/news/world/articles/2020-03-18/el-salvador-registers-first-coronavirus-infection-president-says> |
| 54 | Equatorial Guinea | 14-03-2020 | 16-03-2020 | <https://en.wikipedia.org/wiki/2020_coronavirus_pandemic_in_Africa> | |
| 55 | Estonia | 27-02-2020 | 16-03-2020 | <https://en.unesco.org/themes/education-emergencies/coronavirus-school-closures> | <https://en.wikipedia.org/wiki/2020_coronavirus_pandemic_in_Estonia> |
| 56 | Ethiopia | 13-03-2020 | 16-03-2020 | <https://en.wikipedia.org/wiki/2020_coronavirus_pandemic_in_Africa> | <https://www.aa.com.tr/en/africa/covid-19-ethiopia-closes-schools-bans-public-events/1767683> |
| 57 | Fiji | 19-03-2020 | 19-03-2020 | <https://en.unesco.org/themes/education-emergencies/coronavirus-school-closures> | |
| 58 | Finland | 28-01-2020 | 18-03-2020 | <https://en.unesco.org/themes/education-emergencies/coronavirus-school-closures> | <https://en.wikipedia.org/wiki/2020_coronavirus_pandemic_in_Finland> |
| 59 | France | 24-01-2020 | 16-03-2020 | <https://en.unesco.org/themes/education-emergencies/coronavirus-school-closures> | <https://en.wikipedia.org/wiki/2020_coronavirus_pandemic_in_France> |
| 60 | Gabon | 12-03-2020 | 16-03-2020 | <https://en.wikipedia.org/wiki/2020_coronavirus_pandemic_in_Africa> | <https://en.unesco.org/themes/education-emergencies/coronavirus-school-closures> |
| 61 | Gambia | 17-03-2020 | 18-03-2020 | <https://en.unesco.org/themes/education-emergencies/coronavirus-school-closures> | <https://www.aa.com.tr/en/africa/gambia-confirms-first-coronavirus-case/1769877> |
| 62 | Georgia | 26-02-2020 | 02-03-2020 | <https://en.wikipedia.org/wiki/2020_coronavirus_pandemic_in_Georgia_(country)> | <https://en.unesco.org/themes/education-emergencies/coronavirus-school-closures> |
| 63 | Germany | 27-01-2020 | 18-03-2020 | <https://en.wikipedia.org/wiki/2020_coronavirus_pandemic_in_Germany> | <https://en.unesco.org/themes/education-emergencies/coronavirus-school-closures> |
| 64 | Ghana | 12-03-2020 | 16-03-2020 | <https://en.wikipedia.org/wiki/2020_coronavirus_pandemic_in_Africa> | <https://en.unesco.org/themes/education-emergencies/coronavirus-school-closures> |
| 65 | Greece | 26-02-2020 | 11-03-2020 | <https://en.unesco.org/themes/education-emergencies/coronavirus-school-closures> | <https://en.wikipedia.org/wiki/2020_coronavirus_pandemic_in_Greece> |
| 66 | Guatemala | 13-03-2020 | 16-03-2020 | <https://en.unesco.org/themes/education-emergencies/coronavirus-school-closures> | <https://en.wikipedia.org/wiki/2020_coronavirus_pandemic_in_Guatemala> |
| 67 | Guinea | 13-03-2020 |  | <https://en.wikipedia.org/wiki/2020_coronavirus_pandemic_in_Africa> | |
| 68 | Guinea-Bissau | No | 17-03-2020 | <https://en.unesco.org/themes/education-emergencies/coronavirus-school-closures> | <https://www.dw.com/pt-002/covid-19-guin%C3%A9-bissau-encerra-fronteiras/a-52823220> |
| 69 | Guyana | 11-03-2020 | 16-03-2020 | <https://www.kaieteurnewsonline.com/2020/03/14/coronavirus-effect-education-ministry-announces-two-week-closure-of-public-schools/> | <https://en.wikipedia.org/wiki/2020_coronavirus_pandemic_in_South_America> |
| 70 | Honduras | 10-03-2020 | 12-03-2020 | <https://en.unesco.org/themes/education-emergencies/coronavirus-school-closures> | <https://en.wikipedia.org/wiki/2020_coronavirus_pandemic_in_Honduras> |
| 71 | Hungary | 04-03-2020 | 16-03-2020 | <https://en.unesco.org/themes/education-emergencies/coronavirus-school-closures> | <https://en.wikipedia.org/wiki/2020_coronavirus_pandemic_in_Hungary> |
| 72 | Iceland | 28-02-2020 | 16-03-2020 | <https://en.unesco.org/themes/education-emergencies/coronavirus-school-closures> | <https://en.wikipedia.org/wiki/2020_coronavirus_pandemic_in_Iceland> |
| 73 | India | 30-01-2020 | 20-03-2020 | <https://en.unesco.org/themes/education-emergencies/coronavirus-school-closures> | <https://en.wikipedia.org/wiki/2020_coronavirus_pandemic_in_India> |
| 74 | Indonesia | 02-03-2020 | 16-03-2020 | <https://www.theguardian.com/world/2020/mar/02/first-coronavirus-cases-confirmed-in-indonesia-amid-fears-nation-is-ill-prepared-for-outbreak> | Local |
| 75 | Iran (Islamic Republic of) | 19-02-2020 | 26-02-2020 | <https://en.unesco.org/themes/education-emergencies/coronavirus-school-closures> | <https://en.wikipedia.org/wiki/2020_coronavirus_pandemic_in_Iran> |
| 76 | Iraq | 22-02-2020 | 08-03-2020 | <https://en.unesco.org/themes/education-emergencies/coronavirus-school-closures> | <https://en.wikipedia.org/wiki/2020_coronavirus_pandemic_in_Iraq> |
| 77 | Ireland | 27-02-2020 | 12-03-2020 | <https://en.unesco.org/themes/education-emergencies/coronavirus-school-closures> | <https://en.wikipedia.org/wiki/2020_coronavirus_pandemic_in_Northern_Ireland> |
| 78 | Israel | 21-02-2020 | 12-03-2020 | <https://en.unesco.org/themes/education-emergencies/coronavirus-school-closures> | <https://en.wikipedia.org/wiki/2020_coronavirus_pandemic_in_Israel> |
| 79 | Italy | 31-01-2020 | 04-03-2020 | <https://en.unesco.org/themes/education-emergencies/coronavirus-school-closures> | <https://en.wikipedia.org/wiki/2020_coronavirus_pandemic_in_Italy> |
| 80 | Jamaica | 10-03-2020 | 13-03-2020 | <https://en.unesco.org/themes/education-emergencies/coronavirus-school-closures> | <https://dominicanewsonline.com/news/homepage/homepage-carousel/jamiaca-confirms-first-imported-case-of-covid-19/> |
| 81 | Japan | 16-01-2020 | 12-03-2020 | <https://en.unesco.org/themes/education-emergencies/coronavirus-school-closures> | <https://en.wikipedia.org/wiki/2020_coronavirus_pandemic_in_Japan> |
| 82 | Jordan | 02-03-2020 | 15-03-2020 | <https://en.unesco.org/themes/education-emergencies/coronavirus-school-closures> | <https://en.wikipedia.org/wiki/2020_coronavirus_pandemic_in_Jordan> |
| 83 | Kazakhstan | 12-03-2020 | 16-03-2020 | <https://en.unesco.org/themes/education-emergencies/coronavirus-school-closures> | <https://en.wikipedia.org/wiki/2020_coronavirus_pandemic_in_Kazakhstan> |
| 84 | Kenya | 11-03-2020 | 16-03-2020 | <https://en.wikipedia.org/wiki/2020_coronavirus_pandemic_in_Africa> | <https://www.aa.com.tr/en/africa/coronavirus-kenya-shuts-schools-as-new-cases-confirmed/1767139> |
| 85 | Kiribati |  |  |  |  |
| 86 | Kuwait | 24-02-2020 | 02-03-2020 | <https://www.france24.com/en/20200224-kuwait-bahrain-announce-first-coronavirus-cases> | https://en.unesco.org/themes/education-emergencies/coronavirus-school-closures |
| 87 | Kyrgyzstan | 18-03-2020 | 16-03-2020 | <https://en.wikipedia.org/wiki/2020_coronavirus_pandemic_in_Kyrgyzstan> | https://en.unesco.org/themes/education-emergencies/coronavirus-school-closures |
| 88 | Lao People's Democratic Republic | No | 19-03-2020 | <https://foreignpolicy.com/2020/03/20/laos-coronavirus-free-claims-probably-fiction/> | https://en.unesco.org/themes/education-emergencies/coronavirus-school-closures |
| 89 | Latvia | 02-03-2020 | 13-03-2020 | <https://en.wikipedia.org/wiki/2020_coronavirus_pandemic_in_Latvia> | https://en.unesco.org/themes/education-emergencies/coronavirus-school-closures |
| 90 | Lebanon | 21-02-2020 | 02-03-2020 | <https://en.wikipedia.org/wiki/2020_coronavirus_pandemic_in_Lebanon> | https://en.unesco.org/themes/education-emergencies/coronavirus-school-closures |
| 91 | Lesotho | No | 19-03-2020 | <https://en.wikipedia.org/wiki/2020_coronavirus_pandemic_in_Africa> | https://en.unesco.org/themes/education-emergencies/coronavirus-school-closures |
| 92 | Liberia | 11-03-2020 |  |  |  |
| 93 | Libya | No | 16-03-2020 | <https://en.unesco.org/themes/education-emergencies/coronavirus-school-closures> | <https://en.wikipedia.org/wiki/2020_coronavirus_pandemic_in_Africa> |
| 94 | Lithuania | 28-02-2020 | 16-03-2020 | <https://en.unesco.org/themes/education-emergencies/coronavirus-school-closures> | <https://en.wikipedia.org/wiki/2020_coronavirus_pandemic_in_Lithuania> |
| 95 | Luxembourg | 29-02-2020 | 16-03-2020 | <https://www.aa.com.tr/en/europe/belgium-portugal-to-shut-schools-over-coronavirus/1764338> | <https://en.wikipedia.org/wiki/2020_coronavirus_pandemic_in_Luxembourg> |
| 96 | Madagascar | 20-03-2020 |  | <https://en.wikipedia.org/wiki/2020_coronavirus_pandemic_in_Africa> | |
| 97 | Malawi |  | 20-03-2020 | <https://www.nyasatimes.com/mutharika-lays-out-malawi-response-plan-on-coronavirus-bans-gatherings-of-100-people-schools-closing/> | |
| 98 | Malaysia | 25-01-2020 | 18-03-2020 | https://en.unesco.org/themes/education-emergencies/coronavirus-school-closures | <https://en.wikipedia.org/wiki/2020_coronavirus_pandemic_in_Malaysia> |
| 99 | Maldives | 08-03-2020 | 11-03-2020 | <https://www.straitstimes.com/asia/south-asia/coronavirus-maldives-confirms-first-two-cases-two-islands-locked-down> | <https://en.wikipedia.org/wiki/2020_coronavirus_pandemic_in_the_Maldives#cite_note-3> |
| 100 | Mali |  |  |  |  |
| 101 | Malta | 07-03-2020 | 12-03-2020 | <https://en.wikipedia.org/wiki/2020_coronavirus_pandemic_in_Malta> | <https://timesofmalta.com/articles/view/coronavirus-schools-childcare-centres-university-to-shut-down-for-a.777521> |
| 102 | Marshall Islands | |  |  |  |
| 103 | Mauritania | 13-03-2020 | 16-03-2020 | <https://en.wikipedia.org/wiki/2020_coronavirus_pandemic_in_Africa> | |
| 104 | Mauritius | 19-03-2020 | 19-03-2020 | <https://en.wikipedia.org/wiki/2020_coronavirus_pandemic_in_Africa> | |
| 105 | Mexico | 23-02-2020 | 20-03-2020 | <https://en.wikipedia.org/wiki/2020_coronavirus_pandemic_in_Mexico> | <https://www.marca.com/claro-mx/trending/2020/03/14/5e6d4751268e3eee678b45a3.html> |
| 106 | Micronesia (Federated States of) | No |  | <https://www.rnz.co.nz/international/pacific-news/411999/coronavirus-two-more-cases-confirmed-in-guam> | |
| 107 | Mongolia | 10-03-2020 | 19-03-2020 | https://en.unesco.org/themes/education-emergencies/coronavirus-school-closures | <https://www.garda.com/crisis24/news-alerts/321051/mongolia-first-covid-19-case-confirmed-march-10-update-6> |
| 108 | Montenegro | 17-03-2020 | 16-03-2020 | https://en.unesco.org/themes/education-emergencies/coronavirus-school-closures | <https://nationalpost.com/pmn/health-pmn/montenegro-reports-first-case-of-corona-virus-infection> |
| 109 | Morocco | 02-03-2020 | 16-03-2020 | <https://en.wikipedia.org/wiki/2020_coronavirus_pandemic_in_Africa> | https://en.unesco.org/themes/education-emergencies/coronavirus-school-closures |
| 110 | Mozambique | 22-03-2020 | 22-03-2020 | https://nationalpost.com/pmn/health-pmn/mozambique-confirms-first-coronavirus-case | https://en.unesco.org/themes/education-emergencies/coronavirus-school-closures |
| 111 | Myanmar | 22-03-2020 | 20-03-2020 | https://en.unesco.org/themes/education-emergencies/coronavirus-school-closures | https://www.bangkokpost.com/world/1884820/myanmar-confirms-first-coronavirus-cases |
| 112 | Namibia | 14-03-2020 | 16-03-2020 | <https://en.wikipedia.org/wiki/2020_coronavirus_pandemic_in_Africa> | |
| 113 | Nepal | 24-01-2020 | 18-03-2020 | https://en.unesco.org/themes/education-emergencies/coronavirus-school-closures | <https://en.wikipedia.org/wiki/2020_coronavirus_pandemic_in_Nepal> |
| 114 | Netherlands | 27-02-2020 | 16-03-2020 | https://en.unesco.org/themes/education-emergencies/coronavirus-school-closures | <https://en.wikipedia.org/wiki/2020_coronavirus_pandemic_in_the_Netherlands> |
| 115 | New Zealand | 26-02-2020 | Open | <https://en.wikipedia.org/wiki/2020_coronavirus_pandemic_in_Oceania#Papua_New_Guinea> | |
| 116 | Nicaragua | 19-03-2020 | No | <https://www.elcomercio.com/actualidad/nicaragua-desbordan-supermercados-coronavirus-covid19.html> | <https://confidencial.com.ni/daniel-ortega-ausente-ante-amenaza-coronavirus-nicaragua/> |
| 117 | Niger | 19-03-2020 | DK | <https://en.wikipedia.org/wiki/2020_coronavirus_pandemic_in_Africa> | |
| 118 | Nigeria | 27-02-2020 | 19-03-2020 | <https://en.wikipedia.org/wiki/2020_coronavirus_pandemic_in_Africa> | <https://www.premiumtimesng.com/news/top-news/382806-coronavirus-nigerian-govt-orders-closure-of-schools-nationwide.html> |
| 119 | Niue | No |  |  | <https://en.wikipedia.org/wiki/2020_coronavirus_pandemic_in_Oceania#Papua_New_Guinea> |
| 120 | Norway | 26-02-2020 | 12-03-2020 | <https://en.wikipedia.org/wiki/2020_coronavirus_pandemic_in_Norway> | https://en.unesco.org/themes/education-emergencies/coronavirus-school-closures |
| 121 | Oman | 24-02-2020 |  | <https://en.wikipedia.org/wiki/2020_coronavirus_pandemic_in_Oman> | |
| 122 | Pakistan | 26-02-2020 | 14-03-2020 | https://en.unesco.org/themes/education-emergencies/coronavirus-school-closures | <https://en.wikipedia.org/wiki/2020_coronavirus_pandemic_in_Pakistan> |
| 123 | Palau |  |  |  |  |
| 124 | Palestine | 05-03-2020 | 16-03-2020 | https://en.unesco.org/themes/education-emergencies/coronavirus-school-closures | <https://en.wikipedia.org/wiki/2020_coronavirus_pandemic_in_the_State_of_Palestine> |
| 125 | Panama | 11-03-2020 | 12-03-2020 | https://en.unesco.org/themes/education-emergencies/coronavirus-school-closures | <https://www.jpost.com/Breaking-News/Panama-confirms-first-coronavirus-case-after-woman-showed-symptoms-620387> |
| 126 | Papua New Guinea | 20-03-2020 |  | <https://en.wikipedia.org/wiki/2020_coronavirus_pandemic_in_Oceania#Papua_New_Guinea> | |
| 127 | Paraguay | 07-03-2020 | 11-03-2020 | https://en.unesco.org/themes/education-emergencies/coronavirus-school-closures | https://en.wikipedia.org/wiki/2020_coronavirus_pandemic_in_South_America |
| 128 | Peru | 06-03-2020 | 16-03-2020 | https://en.unesco.org/themes/education-emergencies/coronavirus-school-closures | https://en.wikipedia.org/wiki/2020_coronavirus_pandemic_in_South_America |
| 129 | Philippines | 30-01-2020 | 10-03-2020 | https://en.unesco.org/themes/education-emergencies/coronavirus-school-closures | <https://en.wikipedia.org/wiki/2020_coronavirus_pandemic_in_the_Philippines> |
| 130 | Poland | 04-03-2020 | 13-03-2020 | https://en.unesco.org/themes/education-emergencies/coronavirus-school-closures | <https://en.wikipedia.org/wiki/2020_coronavirus_pandemic_in_Poland> |
| 131 | Portugal | 02-03-2020 | 16-03-2020 | https://en.unesco.org/themes/education-emergencies/coronavirus-school-closures | <https://en.wikipedia.org/wiki/2020_coronavirus_pandemic_in_Portugal> |
| 132 | Qatar | 27-01-2020 | 10-03-2020 | https://en.unesco.org/themes/education-emergencies/coronavirus-school-closures | <https://en.wikipedia.org/wiki/2020_coronavirus_pandemic_in_Qatar> |
| 133 | Republic of Korea | 20-01-2020 | 02-03-2020 | https://en.unesco.org/themes/education-emergencies/coronavirus-school-closures | |
| 134 | Republic of Moldova | 25-02-2020 | 11-03-2020 | https://en.unesco.org/themes/education-emergencies/coronavirus-school-closures | <https://en.wikipedia.org/wiki/2020_coronavirus_pandemic_in_Moldova> |
| 135 | Romania | 26-02-2020 | 11-03-2020 | https://en.unesco.org/themes/education-emergencies/coronavirus-school-closures | <https://en.wikipedia.org/wiki/2020_coronavirus_pandemic_in_Romania> |
| 136 | Russian Federation | 31-01-2020 | 11-03-2020 | https://en.unesco.org/themes/education-emergencies/coronavirus-school-closures | Local |
| 137 | Rwanda | 14-03-2020 | 16-03-2020 | https://en.unesco.org/themes/education-emergencies/coronavirus-school-closures | <https://en.wikipedia.org/wiki/2020_coronavirus_pandemic_in_Africa> |
| 138 | Saint Kitts and Nevis | | 16-03-2020 | https://en.unesco.org/themes/education-emergencies/coronavirus-school-closures | |
| 139 | Saint Lucia | 13-03-2020 | 16-03-2020 | https://en.unesco.org/themes/education-emergencies/coronavirus-school-closures | <https://en.wikipedia.org/wiki/2020_coronavirus_pandemic_in_Saint_Lucia> |
| 140 | Saint Vincent and the Grenadines | 11-03-2020 | 16-03-2020 | https://en.unesco.org/themes/education-emergencies/coronavirus-school-closures | <https://en.wikipedia.org/wiki/2020_coronavirus_pandemic_in_Saint_Vincent_and_the_Grenadines> |
| 141 | Samoa | 19-03-2020 | 23-03-2020 | <https://www.thenewhumanitarian.org/news/2020/03/20/samoa-emergency-coronavirus> | <https://www.tvnz.co.nz/one-news/world/tonga-samoa-both-announce-states-emergency-in-response-coronavirus-pandemic> |
| 142 | San Marino | 27-02-2020 |  | <https://en.wikipedia.org/wiki/2020_coronavirus_pandemic_in_San_Marino> | |
| 143 | Sao Tome and Principe | No | 20-03-2020 | https://en.unesco.org/themes/education-emergencies/coronavirus-school-closures | <https://en.wikipedia.org/wiki/2020_coronavirus_pandemic_in_Africa> |
| 144 | Saudi Arabia | 02-03-2020 | 09-03-2020 | https://en.unesco.org/themes/education-emergencies/coronavirus-school-closures | <https://en.wikipedia.org/wiki/2020_coronavirus_pandemic_in_Saudi_Arabia> |
| 145 | Senegal | 02-03-2020 | 16-03-2020 | https://en.unesco.org/themes/education-emergencies/coronavirus-school-closures | <https://en.wikipedia.org/wiki/2020_coronavirus_pandemic_in_Africa> |
| 146 | Serbia | 06-03-2020 | 16-03-2020 | https://en.unesco.org/themes/education-emergencies/coronavirus-school-closures | <https://en.wikipedia.org/wiki/2020_coronavirus_pandemic_in_Serbia> |
| 147 | Seychelles | 14-03-2020 | 16-03-2020 | https://en.unesco.org/themes/education-emergencies/coronavirus-school-closures | <https://en.wikipedia.org/wiki/2020_coronavirus_pandemic_in_Africa> |
| 148 | Sierra Leone | No |  |  |  |
| 149 | Singapore | 23-01-2020 | No | <https://www.theguardian.com/education/2020/mar/17/schools-across-england-struggle-as-coronavirus-hits-attendance> | <https://en.wikipedia.org/wiki/2020_coronavirus_pandemic_in_Singapore> |
| 150 | Slovakia | 06-03-2020 | 16-03-2020 | https://en.unesco.org/themes/education-emergencies/coronavirus-school-closures | <https://en.wikipedia.org/wiki/2020_coronavirus_pandemic_in_Slovakia> |
| 151 | Slovenia | 04-03-2020 | 16-03-2020 | https://en.unesco.org/themes/education-emergencies/coronavirus-school-closures | <https://en.wikipedia.org/wiki/2020_coronavirus_pandemic_in_Slovenia> |
| 152 | Solomon Islands | |  |  |  |
| 153 | Somalia | 16-03-2020 | 18-03-2020 | https://en.unesco.org/themes/education-emergencies/coronavirus-school-closures | <https://en.wikipedia.org/wiki/2020_coronavirus_pandemic_in_Africa> |
| 154 | South Africa | 05-03-2020 | 18-03-2020 | https://en.unesco.org/themes/education-emergencies/coronavirus-school-closures | <https://en.wikipedia.org/wiki/2020_coronavirus_pandemic_in_Africa> |
| 155 | Spain | 31-01-2020 | 16-03-2020 | https://en.unesco.org/themes/education-emergencies/coronavirus-school-closures | <https://en.wikipedia.org/wiki/2020_coronavirus_pandemic_in_Spain> |
| 156 | Sri Lanka | 27-01-2020 | 13-03-2020 | https://en.unesco.org/themes/education-emergencies/coronavirus-school-closures | <https://en.wikipedia.org/wiki/2020_coronavirus_pandemic_in_Sri_Lanka> |
| 157 | Sudan | 13-03-2020 | 16-03-2020 |  | <https://en.wikipedia.org/wiki/2020_coronavirus_pandemic_in_Africa> |
| 158 | Suriname | 13-03-2020 | No |  | https://en.wikipedia.org/wiki/2020_coronavirus_pandemic_in_South_America |
| 159 | Swaziland | 14-03-2020 | No | <https://www.iol.co.za/news/africa/eswatini-confirms-first-coronavirus-case-44862050> | |
| 160 | Sweden | 31-01-2020 | 18-03-2020 | https://en.unesco.org/themes/education-emergencies/coronavirus-school-closures | <https://sverigesradio.se/sida/artikel.aspx?programid=2054&artikel=7398979> |
| 161 | Switzerland | 23-02-2020 | 16-03-2020 | https://en.unesco.org/themes/education-emergencies/coronavirus-school-closures | <https://en.wikipedia.org/wiki/2020_coronavirus_pandemic_in_Switzerland> |
| 162 | Syrian Arab Republic | No | 16-03-2020 | https://en.unesco.org/themes/education-emergencies/coronavirus-school-closures | <https://timesofindia.indiatimes.com/world/middle-east/war-ravaged-syria-takes-new-steps-against-coronavirus-says-no-recorded-cases-yet/articleshow/74732588.cms> |
| 163 | Taiwan | 21-01-2020 | 20-02-2020 | <https://www.taiwannews.com.tw/en/news/3878803> | |
| 164 | Tajikistan | No |  | <https://www.rferl.org/a/tajik-workers-face-dire-future-as-russia-closes-borders-over-coronavirus/30495815.html> | |
| 165 | Thailand | 16-01-2020 | 18-03-2020 | https://en.unesco.org/themes/education-emergencies/coronavirus-school-closures | <https://en.wikipedia.org/wiki/2020_coronavirus_pandemic_in_Thailand> |
| 166 | The former Yugoslav Republic of Macedonia | 26-02-2020 | 11-03-2020 | https://en.unesco.org/themes/education-emergencies/coronavirus-school-closures | <https://en.wikipedia.org/wiki/2020_coronavirus_pandemic_in_North_Macedonia> |
| 167 | Timor-Leste | |  |  |  |
| 168 | Togo | 06-03-2020 | 20-03-2020 | <https://l-frii.com/coronavirus-le-togo-ferme-toutes-les-ecoles-primaires-secondaires-et-professionnelles/> | <https://en.wikipedia.org/wiki/2020_coronavirus_pandemic_in_Africa> |
| 169 | Tonga | 04-03-2020 |  |  | <https://www.rnz.co.nz/international/pacific-news/410994/coronavirus-4-in-isolation-in-fiji-1-suspected-case-in-tonga> |
| 170 | Trinidad and Tobago | 12-03-2020 | 14-03-2020 | https://en.unesco.org/themes/education-emergencies/coronavirus-school-closures | <https://en.wikipedia.org/wiki/2020_coronavirus_pandemic_in_Trinidad_and_Tobago> |
| 171 | Tunisia | 02-03-2020 | 16-03-2020 | https://en.unesco.org/themes/education-emergencies/coronavirus-school-closures | <https://en.wikipedia.org/wiki/2020_coronavirus_pandemic_in_Africa> |
| 172 | Turkey | 11-03-2020 | 12-03-2020 | https://en.unesco.org/themes/education-emergencies/coronavirus-school-closures | <https://english.alarabiya.net/en/News/middle-east/2020/03/12/Turkey-to-close-schools-universities-for-a-week-over-coronavirus.html> |
| 173 | Uganda | No | 20-03-2020 | https://en.unesco.org/themes/education-emergencies/coronavirus-school-closures | <https://health.go.ug/press-release> |
| 174 | Ukraine | 03-03-2020 | 12-03-2020 | https://en.unesco.org/themes/education-emergencies/coronavirus-school-closures | <https://en.wikipedia.org/wiki/2020_coronavirus_pandemic_in_Ukraine> |
| 175 | United Arab Emirates | 29-01-2020 | 08-03-2020 | https://en.unesco.org/themes/education-emergencies/coronavirus-school-closures | <https://en.wikipedia.org/wiki/2020_coronavirus_pandemic_in_the_United_Arab_Emirates> |
| 176 | United Kingdom | 31-01-2020 | 20-03-2020 | https://en.unesco.org/themes/education-emergencies/coronavirus-school-closures | <https://en.wikipedia.org/wiki/2020_coronavirus_pandemic_in_the_United_Kingdom> |
| 177 | United Republic of Tanzania | 16-03-2020 | 19-03-2020 | https://en.unesco.org/themes/education-emergencies/coronavirus-school-closures | <https://en.wikipedia.org/wiki/2020_coronavirus_pandemic_in_Africa> |
| 178 | United States of America | 24-01-2020 |  | <https://www.worldometers.info/coronavirus/usa-coronavirus/> | |
| 179 | Uruguay | 13-03-2020 | 14-03-2020 | https://en.unesco.org/themes/education-emergencies/coronavirus-school-closures | https://en.wikipedia.org/wiki/2020_coronavirus_pandemic_in_South_America |
| 180 | Uzbekistan | 15-03-2020 | 16-03-2020 | https://en.unesco.org/themes/education-emergencies/coronavirus-school-closures | <https://en.wikipedia.org/wiki/2020_coronavirus_pandemic_in_Uzbekistan> |
| 181 | Vanuatu | No | No | <https://www.usnews.com/news/world/articles/2020-03-18/vanuatu-to-impose-coronavirus-travel-restrictions-ahead-of-election> | |
| 182 | Venezuela (Bolivarian Republic of) | 13-03-2020 | 16-03-2020 | https://en.unesco.org/themes/education-emergencies/coronavirus-school-closures | https://en.wikipedia.org/wiki/2020_coronavirus_pandemic_in_South_America |
| 183 | Viet Nam | 23-01-2020 | 28-02-2020 | <https://en.wikipedia.org/wiki/2020_coronavirus_pandemic_in_Vietnam> | https://en.unesco.org/themes/education-emergencies/coronavirus-school-closures |
| 184 | Yemen | No | 19-03-2020 | <https://www.aljazeera.com/news/2020/03/yemen-suspends-flights-closes-schools-coronavirus-fears-200319105953270.html> | <https://theintercept.com/2020/03/18/yemens-health-care-coronavirus-covid-19/> |
| 185 | Zambia | 18-03-2020 | 20-03-2020 | <https://en.unesco.org/themes/education-emergencies/coronavirus-school-closures> | <https://en.wikipedia.org/wiki/2020_coronavirus_pandemic_in_Africa> |
| 186 | Zimbabwe | 15-03-2020 | 24-03-2020 | <https://www.herald.co.zw/president-orders-schools-colleges-to-close-next-week/> | <https://en.wikipedia.org/wiki/2020_coronavirus_pandemic_in_Zimbabwe> |
