# Supplementary material for: The global viralization of policies to contain the spreading of the COVID-19 pandemic: Analyses of school closures and first reported cases
Source: PLoS One. 2021 Apr 1;16(4):e0248828. doi: 10.1371/journal.pone.0248828 (PMC8016240; doi:10.1371/journal.pone.0248828)
Supplement: S2 File — (DOCX) [file pone.0248828.s002.docx]

**S2 File**

List with United Nations regional clusters and countries

This list is based on United Nations’ classification (United Nations 2015).

Africa

1. Eastern Africa:

Burundi, Comoros, Djibouti, Eritrea, Ethiopia, Kenya, Madagascar, Zambia, Zimbabwe, Malawi, Mayotte, Mozambique, Rwanda, Seychelles, South Sudan, Somalia, Uganda, United Republic of Tanzania and Mauritius

2. Middle Africa:

Angola, Congo, Cameroon, Central African Republic, Chad, Democratic Republic of the Congo, Equatorial Guinea, Sao Tome and Principe, and Gabon

3. Northern Africa:

Algeria, Egypt, Libya, Morocco, Sudan, and Tunisia

4. Western Africa:

Benin, Burkina Faso, Cabo Verde, Cote de Ivoire, Gambia, Ghana, Guinea, Guinea-Bissau, Liberia, Mali, Mauritania, Niger, Nigeria, Senegal, Sierra Leone, and Togo

5. Southern Africa:

Botswana, Lesotho, Namibia, South Africa, and Swaziland

Asia

6. Eastern Asia

China, Japan, Taiwan, Republic of Korea, Democratic People's Republic of Korea, and Mongolia

7. Central Asia:

Kazakhstan, Kyrgyzstan, Tajikistan, Turkmenistan, and Uzbekistan

8. Southern Asia

Afghanistan, Bangladesh, Bhutan, India, Iran (Islamic Republic of), Maldives, Nepal, Pakistan, and Sri Lanka

9. South-Eastern Asia

Brunei Darussalam, Cambodia, Indonesia, Lao People's Democratic Republic, Malaysia, Myanmar, Philippines, Singapore, Thailand, Timor-Leste, and Viet Nam.

10. Western Asia

Armenia, Azerbaijan, Bahrain, Cyprus, Georgia, Iraq, Israel, Jordan, Kuwait, Lebanon, Oman, Qatar, Saudi Arabia, Syrian Arab Republic, Turkey, United Arab Emirates, and Yemen

Europe

11. Eastern Europe

Belarus, Bulgaria, Czech Republic, Hungary, Poland, Republic of Moldova, Romania, Russian Federation, Slovakia, and Ukraine

12. Northern Europe

Denmark, Estonia, Finland, Iceland, Latvia, Lithuania, Norway, Sweden, Northern Ireland, Ireland, and United Kingdom

13. Southern Europe

Albania, Andorra, Bosnia and Herzegovina, Croatia, Greece, Italy, Malta, Montenegro, Portugal, San Marino, Serbia, Slovenia, Spain, The former Yugoslav Republic of Macedonia

14. Western Europe

Austria, Belgium, France, Germany, Liechtenstein, Luxembourg, Monaco, Netherlands, and Switzerland

America

15. Caribbean

Antigua and Barbuda, Bahamas, Barbados, Cayman Islands, Cuba, Dominica, Dominican Republic, Grenada, Haiti, Jamaica, Martinique, Puerto Rico, Saint Kitts and Nevis, Saint Lucia, Saint Vincent and the Grenadines and Trinidad and Tobago

16. Central America

Belize, Costa Rica, El Salvador, Guatemala, Honduras, Mexico, Nicaragua, and Panama

17. South America

Argentina, Bolivia (Plurinational State of), Brazil, Chile, Colombia, Ecuador, Guyana, French Guiana, Paraguay, Peru, Suriname, Uruguay, and Venezuela (Bolivarian Republic of).

18. Northern America

Canada, United States of America, and Bermuda

Oceania

19. Australia/New Zealand

Australia and New Zealand

20. Melanesia

Fiji, Papua New Guinea, Solomon Islands, and Vanuatu

21. Micronesia

Guam, Kiribati, Micronesia (Federated States of), Nauru, Palau and Marshall Islands

22. Polynesia

Cook Islands, Niue, Samoa, Tokelau, Tonga, and Tuvalu
