# Supplementary material for: The global viralization of policies to contain the spreading of the COVID-19 pandemic: Analyses of school closures and first reported cases
Source: PLoS One. 2021 Apr 1;16(4):e0248828. doi: 10.1371/journal.pone.0248828 (PMC8016240; doi:10.1371/journal.pone.0248828)
Supplement: S3 File — (DOCX) [file pone.0248828.s003.docx]

**S3 File.** Analysis with Gompertz and Exponential

**S3.1 Table** Gompertz models predicting first reported case of COVID-19 and school closure

|  | First reported case of COVID-19  *Onset of risk: December 31st, 2019-China reports to WHO’s authorities the epidemic in Wuhan* | | | First reported case of COVID-19  *Onset of risk: January 31st, 2020-WHO declares global health emergency* | | | Date in which schools were closed at the national level  *Onset of risk: December 31st, 2019-China reports to WHO’s authorities the epidemic in Wuhan* | | | Date in which schools were closed at the national level*.*  *Onset of risk: January 31st, 2020-WHO declares global health emergency* | | | Date in which schools were closed at the national level*.*  *Respective date a country reports its first case of COVID-19* | | |
| --- | --- | --- | --- | --- | --- | --- | --- | --- | --- | --- | --- | --- | --- | --- | --- |
| Independent Variables | Hazard Ratio | 95% CI | | Hazard Ratio | 95% CI | | Hazard Ratio | 95% CI | | Hazard Ratio | 95% CI | | Hazard Ratio | 95% CI | |
| *Epidemic security index (z score)* | 1.34 | 1.04 | 1.73 | 1.39 | 1.09 | 1.0276 | 0.63 | 0.51 | 0.79 | 0.63 | 0.51 | 0.79 | 0.63 | 0.51 | 0.79 |
| *GDP per capita (ln)* | 1.65 | 1.32 | 2.29 | 1.70 | 1.24 | 2..28 | 0.94 | 0.72 | 1.22 | 0.94 | 0.72 | 1.22 | 0.91 | 0.70 | 1.17 |
| *Population size (ln)* | 1..34 | 1.22 | 1.64 | 1.27 | 1.06 | 1.52 | 1.14 | 0.94 | 1.39 | 1.14 | 0.94 | 1.39 | 1.06 | 0.90 | 1.26 |
| *Democracy (z score)* |  |  |  |  |  |  | 0.69 | 0.43 | 1.11 | 0.69 | 0.43 | 1.11 | 0.69 | 0.44 | 1.07 |
| *Globalization index (z score)* | 1.28 | 0.83 | 1.97 | 1.35 | 0.82 | 2.20 | 2.11 | 1.46 | 3.05 | 2.10 | 1.46 | 3.03 | 1.75 | 1.35 | 2.26 |
| *Economic Integration to South Korea* | 1.00 | 1.00 | 1.00 | 1.00 | 0.99 | 1.00 | 0.99 | 0.99 | 1.00 | 0.99 | 0.99 | 1.00 | 0.99 | 0.99 | 1.00 |
| *Economic Integration to Italy* | 0.99 | 0.99 | 1.00 | 0.99 | 0.99 | 1.00 | 1.00 | 1.00 | 1.00 | 1.00 | 1.00 | 1.00 | 1.00 | 1.00 | 1.00 |
|  |  |  |  |  |  |  |  |  |  |  |  |  |  |  |  |
|  |  |  |  |  |  |  |  |  |  |  |  |  |  |  |  |
| *Number of countries* |  | 165 |  |  | 143 |  |  | 142 |  |  | 142 |  |  | 127 |  |
| *Number of adoptions* |  | 165 |  |  | 143 |  |  | 138 |  |  | 138 |  |  | 123 |  |
| *Time at risk* | 10411 | | |  | 5404 |  |  | 10875 |  |  | 6473 |  |  | 2100 |  |

All models adjusted for clustering at the region level. CI Confidence Interval.

**S3.2 Table** Exponential models predicting first reported case of COVID-19 and school closure

|  | First reported case of COVID-19  *Onset of risk: December 31st, 2019-China reports to WHO’s authorities the epidemic in Wuhan* | | | First reported case of COVID-19  *Onset of risk: January 31st, 2020-WHO declares global health emergency* | | | Date in which schools were closed at the national level  *Onset of risk: December 31st, 2019-China reports to WHO’s authorities the epidemic in Wuhan* | | | Date in which schools were closed at the national level*.*  *Onset of risk: January 31st, 2020-WHO declares global health emergency* | | | Date in which schools were closed at the national level*.*  *Respective date a country reports its first case of COVID-19* | | |
| --- | --- | --- | --- | --- | --- | --- | --- | --- | --- | --- | --- | --- | --- | --- | --- |
| Independent Variables | Hazard Ratio | 95% CI | | Hazard Ratio | 95% CI | | Hazard Ratio | 95% CI | | Hazard Ratio | 95% CI | | Hazard Ratio | 95% CI | |
| *Epidemic security index (z score)* | 1.07 | 1.02 | 1.11 | 1.03 | 0.98 | 1.07 | 0.97 | 0.93 | 1.01 | 0.96 | 0.91 | 1.00 | 0.73 | 0.60 | 0.90 |
| *GDP per capita (ln)* | 1.12 | 1.07 | 1.18 | 1.13 | 1.07 | 1.19 | 1.01 | 0.97 | 1.05 | 1.03 | 0.93 | 1.07 | 0.82 | 0.66 | 1.01 |
| *Population size (ln)* | 1.09 | 1.05 | 1.12 | 1.07 | 1.04 | 1.10 | 1.02 | 0.96 | 1.05 | 1.02 | 1.00 | 1.05 | 0.82 | 0.75 | 0.91 |
| *Democracy (z score)* |  |  |  |  |  |  | 0.96 | 0.87 | 1.06 | 0.93 | 0.99 | 1.05 | 0.92 | 0.69 | 1.01 |
| *Globalization index (z score)* | 1.03 | 0.93 | 1.15 | 1.00 | 0.99 | 1.01 | 1.04 | 0.98 | 1.12 | 1.07 | 1.00 | 1.16 | 1.04 | 0.68 | 1.04 |
| *Economic Integration to South Korea* | 1.00 | 0.99 | 1.00 | 1.00 | 0.99 | 1.00 | 1.00 | 0.99 | 1.00 | 1.00 | 0.99 | 1.00 | 1.00 | 0.99 | 1.00 |
| *Economic Integration to Italy* | 0.99 | 0.99 | 1.00 | 1.00 | 0.99 | 1.00 | 1.00 | 0.99 | 1.00 | 1.00 | 0.99 | 1.00 | 1.00 | 0.99 | 1.00 |
|  |  |  |  |  |  |  |  |  |  |  |  |  |  |  |  |
|  |  |  |  |  |  |  |  |  |  |  |  |  |  |  |  |
| *Number of countries* |  | 164 |  |  | 143 |  |  | 156 |  |  | 142 |  |  | 127 |  |
| *Number of adoptions* |  | 164 |  |  | 143 |  |  | 141 |  |  | 138 |  |  | 123 |  |
| *Time at risk* | 10316 | | |  | 5404 |  |  | 12214 |  |  | 6473 |  |  | 2100 |  |

All models adjusted for clustering at the region level. CI Confidence Interval.
