# Supplementary material for: The global viralization of policies to contain the spreading of the COVID-19 pandemic: Analyses of school closures and first reported cases
Source: PLoS One. 2021 Apr 1;16(4):e0248828. doi: 10.1371/journal.pone.0248828 (PMC8016240; doi:10.1371/journal.pone.0248828)
Supplement: S4 File — (DOCX) [file pone.0248828.s004.docx]

**S4 File**

**S4.1 Table** Survival analysis with countries which nationally and using the last date in which countries locally closed schools.

| *Outcome* | Date in which schools were closed at the national level*.* | | | | | | | | |
| --- | --- | --- | --- | --- | --- | --- | --- | --- | --- |
| *Onset* | *December 31st, 2019-China reports to WHO’s authorities the epidemic in Wuhan* | | | *January 31st, 2020-WHO declares global health emergency* | | | *Respective date a country reports its first case of COVID-19* | | |
| *Determinants* | Hazard Ratio | 95% CI | | Hazard Ratio | 95% CI | | Hazard Ratio | 95% CI | |
| *Epidemic security index (z score)* | 0.64 | 0.52 | 0.79 | 0.64 | 0.52 | 0.79 | 0.62 | 0.48 | 0.80 |
| *GDP per capita (ln)* | 0.91 | 0.68 | 1.20 | 0.91 | 0.68 | 1.20 | 0.88 | 0.70 | 1.17 |
| *Population size (ln)* | 1.09 | 0.91 | 1.30 | 1.09 | 0.91 | 1.31 | 1.02 | 0.85 | 1.21 |
| *Democracy (z score)* | 0.73 | 0.46 | 1.17 | 0.73 | 0.46 | 1.17 | 0.74 | 0.47 | 1.17 |
| *Globalization index (z score)* | 2.03 | 1.41 | 2.92 | 2.03 | 1.41 | 2.92 | 1.69 | 1.29 | 2.22 |
| *Economic Integration to South Korea* | 1.00 | 0.99 | 1.00 | 1.00 | 0.99 | 1.00 | 0.99 | 0.99 | 1.00 |
| *Economic Integration to Italy* | 1.00 | 1.00 | 1.00 | 1.00 | 1.00 | 1.00 | 1.00 | 1.00 | 1.00 |
|  |  |  |  |  |  |  |  |  |  |
| *Time (ρ)* | 11.43 | 9.66 | 13.52 | 11.42 | 9.66 | 13.52 | 8.31 | 6.46 | 10.69 |
|  |  |  |  |  |  |  |  |  |  |
| *Number of countries* |  | 149 |  |  | 149 |  |  | 132 |  |
| *Number of adoptions* |  | 142 |  |  | 142 |  |  | 111 |  |
| *Time at risk* |  | 11430 |  |  | 6811 |  |  | 2304 |  |

All models adjusted for clustering at the region level. CI Confidence Interval.
