# Supplementary material for: The global viralization of policies to contain the spreading of the COVID-19 pandemic: Analyses of school closures and first reported cases
Source: PLoS One. 2021 Apr 1;16(4):e0248828. doi: 10.1371/journal.pone.0248828 (PMC8016240; doi:10.1371/journal.pone.0248828)
Supplement: S5 File — (DOCX) [file pone.0248828.s005.docx]

**S5 File**

**S5.1 Table** Negative binomial models predicting ‘Date in which schools were closed at the national level.’

| *Outcome* | Date in which schools were closed at the national level*.* | | | | | | | | |
| --- | --- | --- | --- | --- | --- | --- | --- | --- | --- |
| *Onset* | *December 31st, 2019-China reports to WHO’s authorities the epidemic in Wuhan* | | | *January 31st, 2020-WHO declares global health emergency* | | | *Respective date a country reports its first case of COVID-19* | | |
| *Determinants* | Coef. | 95% CI | | Coef. | 95% CI | | Coef. | 95% CI | |
| *Epidemic security index (z score)* | 0.02 | 0.00 | 0.04 | 0.03 | 0.00 | 0.06 | 0.38 | 0.23 | 0.53 |
| *GDP per capita (ln)* | -0.01 | -0.03 | 0.01 | -0.02 | -0.05 | 0.01 | 0.15 | -0.16 | 0.46 |
| *Population size (ln)* | 0.00 | -0.01 | 0.01 | 0.00 | -0.02 | 0.02 | 0.29 | -0.19 | 0.78 |
| *Democracy (z score)* | 0.04 | 0.00 | 0.07 | 0.07 | 0.01 | 0.13 | 0.00 | -0.27 | 0.27 |
| *Globalization index (z score)* | -0.04 | -0.07 | -0.02 | -0.07 | -0.11 | -0.04 | 0.11 | -0.45 | 0.68 |
| *Economic Integration to South Korea* | 0.00 | 0.00 | 0.00 | 0.00 | 0.00 | 0.00 | 0.00 | 0.00 | 0.00 |
| *Economic Integration to Italy* | 0.00 | 0.00 | 0.00 | 0.00 | 0.00 | 0.00 | 0.00 | 0.00 | 0.00 |
|  |  |  |  |  |  |  |  |  |  |
| *Number of countries* |  | 146 |  |  | 146 |  |  | 143 |  |

**S5.2 Table** Linear regression models predicting ‘Date in which schools were closed at the national level.’

| *Outcome* | Date in which schools were closed at the national level*.* | | | | | | | | |
| --- | --- | --- | --- | --- | --- | --- | --- | --- | --- |
| *Onset* | *December 31st, 2019-China reports to WHO’s authorities the epidemic in Wuhan* | | | *January 31st, 2020-WHO declares global health emergency* | | | *Respective date a country reports its first case of COVID-19* | | |
| *Determinants* | Coef. | 95% CI | | Coef. | 95% CI | | Coef. | 95% CI | |
| *Epidemic security index (z score)* | 1.56 | 0.14 | 2.98 | 1.56 | 0.14 | 2.98 | 4.72 | 1.65 | 7.78 |
| *GDP per capita (ln)* | -0.82 | -2.37 | 0.74 | -0.82 | -2.37 | 0.74 | 3.92 | 0.65 | 7.20 |
| *Population size (ln)* | -0.12 | -1.13 | 0.88 | -0.12 | -1.13 | 0.88 | 4.02 | 2.44 | 5.61 |
| *Democracy (z score)* | 2.96 | 0.09 | 5.82 | 2.96 | 0.09 | 5.82 | 0.31 | -4.40 | 5.02 |
| *Globalization index (z score)* | -3.39 | -5.61 | -1.18 | -3.39 | -5.61 | -1.18 | 0.97 | -7.04 | 8.99 |
| *Economic Integration to South Korea* | 0.00 | 0.00 | 0.00 | 0.00 | 0.00 | 0.00 | 0.01 | 0.00 | 0.01 |
| *Economic Integration to Italy* | 0.00 | -0.01 | 0.00 | 0.00 | -0.01 | 0.00 | -0.01 | -0.02 | 0.00 |
|  |  |  |  |  |  |  |  |  |  |
| *Number of countries* |  | 146 |  |  | 146 |  |  | 143 |  |
