# Supplementary material for: The global viralization of policies to contain the spreading of the COVID-19 pandemic: Analyses of school closures and first reported cases
Source: PLoS One. 2021 Apr 1;16(4):e0248828. doi: 10.1371/journal.pone.0248828 (PMC8016240; doi:10.1371/journal.pone.0248828)
Supplement: S6 File — (DOCX) [file pone.0248828.s006.docx]

**S6 File**

STATA codes

STATA code

stset firstcase1, time0(Chinaexposure1) origin(time Chinaexposure1)

stset firstcase1, time0(globalhealthWHO) origin(time Chinaexposure1)

**Table 1 and tables in the appendix**

foreach model in exponential w llogistic gompertz {

streg RapidResponse GDPPOPLOG logPOP globalization DICHI DITALIA , dist (`model') vce (cluster NetworkUNregion)

estimates store `model'

}

estimates stats _all

stset closeschools12, failure (failureschool)

stset closeschools12, time0(Chinaexposure1) origin(time Chinaexposure1) failure(failureschool)

stset closeschools12, time0(globalhealthWHO) origin(time Chinaexposure1) failure(failureschool==1)

stset closeschools12, time0(OMS) origin(time Chinaexposure1) failure(failureschool==1)

stset closeschools12, time0(firstcase1) origin(time Chinaexposure1) failure(failureschool)

foreach model in exponential weibull llogistic gompertz {

streg RapidResponse GDPPOPLOG logPOP democracyindexeiu globalization DICHI DITALIA, dist (`model') vce (cluster NetworkUNregion)

estimates store `model'

}

estimates stats _all
