# Supplementary material for: The global viralization of policies to contain the spreading of the COVID-19 pandemic: Analyses of school closures and first reported cases
Source: PLoS One. 2021 Apr 1;16(4):e0248828. doi: 10.1371/journal.pone.0248828 (PMC8016240; doi:10.1371/journal.pone.0248828)
Supplement: S7 File — (DOCX) [file pone.0248828.s007.docx]

**S7 File**

**S7.1 Table** Survival analysis with variable ‘Early detection and reporting epidemics of potential international concern.’

| *Outcome* | First reported case of COVID-19 | | | | | |
| --- | --- | --- | --- | --- | --- | --- |
| *Onset* | *December 31st, 2019-China reports to WHO’s authorities the epidemic in Wuhan* | | | *January 31st, 2020-WHO declares global health emergency* | | |
| *Determinants* | Hazard Ratio | 95% CI | | Hazard Ratio | 95% CI | |
| *Early detection and reporting epidemics of potential international concern* | 1.21 | 1.02 | 1.44 | 1.39 | 1.00 | 1.94 |
| *GDP per capita (ln)* | 1.54 | 1.19 | 2.00 | 1.73 | 1.27 | 2.35 |
| *Population size (ln)* | 1.37 | 1.21 | 1.53 | 1.34 | 1.02 | 1.76 |
| *Globalization index (z score)* | 1.35 | 0.90 | 2.03 | 1.51 | 0.84 | 2.70 |
| *Economic Integration to South Korea* | 1.00 | 1.00 | 1.00 | 1.00 | 1.00 | 1.00 |
| *Economic Integration to Italy* | 0.99 | 0.99 | 1.00 | 1.00 | 0.99 | 1.00 |
|  |  |  |  |  |  |  |
| *Time (ρ)* | 5.73 | 3.92 | 8.37 | 10.25 | 7.99 | 13.16 |
|  |  |  |  |  |  |  |
| *Number of countries* |  | 149 |  |  | 127 |  |
| *Number of adoptions* |  | 149 |  |  | 127 |  |
| *Time at risk* | 9231 | | |  | 4720 |  |
