# Supplementary material for: The global viralization of policies to contain the spreading of the COVID-19 pandemic: Analyses of school closures and first reported cases
Source: PLoS One. 2021 Apr 1;16(4):e0248828. doi: 10.1371/journal.pone.0248828 (PMC8016240; doi:10.1371/journal.pone.0248828)
Supplement: S8 File — (DOCX) [file pone.0248828.s008.docx]

**S8 File**

**S8.1 Fig** Speed of reporting first case of COVID-19 with two onsets: i) December 31st, 2019-China reports to WHO’s authorities the epidemic in Wuhan and ii) January 31st, 2020-WHO declares global health emergency

**S8.2 Fig** Speed of closing schools with two onsets: i) January 31st, 2020-WHO declares global health emergency and i) Respective date a country reports its first case of COVID-19
