# Supplementary material for: The global viralization of policies to contain the spreading of the COVID-19 pandemic: Analyses of school closures and first reported cases
Source: PLoS One. 2021 Apr 1;16(4):e0248828. doi: 10.1371/journal.pone.0248828 (PMC8016240; doi:10.1371/journal.pone.0248828)
Supplement: Attachment — Submitted filename: Revision PLOS ONE.docx [file pone.0248828.s009.docx]

**Academic Editor**

1. Explore how else you could make figure 2 more informative.

**Response**

We have deleted Figure 2 since it is not highly informative.

2. Be more explicit about your data collection from multiple sources in 172 countries. Are the sources open repositories? if no, how did you obtain permission to extract data from the other closed sources?

**Response**

We explain that sources are open repositories

**Reviewer 1**

1. Abstract: The abstract is clear and concise. It summarises the article adequately to provide the reader with the essence of the paper. Paragraph 3 of page 2 (line 2 and 3): the width of the CI were missing and requires correction.

**Response**

Thank you very much for this comment. We have updated the Cis.

2. Introduction: This is a research paper that addresses an interesting and relevant area of research. This section illustrates the context of the research topic. The authors presented their plausible hypothesis well. However, the paragraph listing the hypotheses 1.1 to 2.5 is difficult to read. It may be beneficial to use a figure with the list of the hypotheses.

**Response**

Thank you for this suggestion, we have added a table to easily identified each hypothesis.

3. Methods and data: The authors provided the sources of the data with explanations for the choice where necessary. This section presents an adequate description and explanation of the methods used in the study. The authors provided additional measures to ensure reliability and validity. There was rigour demonstrated in the selection of data sources, data collection and the analysis. The readers are informed of the methods used to check for items like heterogenicity, sensitivity test etc.

**Response**

Thank you for this appreciation.

4. Results and discussion: The authors presented the results using figures and tables, which made the reading easy for the reader. The tables were clear and assisting the reader in understanding the numbers. The authors performed statistical analysis appropriately and rigorously.

**Response**

Thank you for this appreciation.

5. The assumptions of the results are explained and supported by raw data. The authors provided all the relevant data underlying the findings of the study as annexures. The study appropriately interpreted the results. The discussion is well written, supported by fitting references.

**Response**

Thank you for this appreciation.

6. Conclusion: This research paper derived its conclusions from the study findings.

**Response**

Thank you for this appreciation

7. References: The paper cited adequate and appropriate references.

**Response**

Thank you for this appreciation

8. General: The research has been conducted rigorously, with the appropriate data source, data collection, analysis and interpretation. The language used is clear and straightforward and easy to understand.

**Response**

Thank you for this appreciation

**Reviewer 2**

1. The authors should consider revising the opening statement of the background of the abstract such that "with" will not be used.

**Response**

Thank you. We have re-written the opening statement considering also what the reviewer mentioned in number 3.

2. The last sentence of the methods section of the abstract appears to be inappropriately placed as it is part of the objective(s) of the study and not methods or approaches to conducting this study.

**Response**

Thank you for brining this to our attention. We have moved this sentence to background.

3. The results section included "Ten days after the World Health Organization (WHO) declared COVID-19 to be an international emergency" It is important to include the exact date that this declaration was made in the background section of the abstract as stated in the introduction section of the manuscript.

**Response**

We have added the date. Indeed, this clarifies the abstract much more.

4. This statement "Ten days after the World Health Organization (WHO) declared COVID-19 to be an international emergency, countries were 28 (95% CI: 12,77) times more likely to report first COVID-19 cases and 42 (95% CI: 22,90) times more likely to close schools" in the results section is unclear as there was no reference for comparison for the measures of effects used, was it as compared 7 days depicted in the results section of the manuscript?

**Response**.

Thank you for brining this to our attention, we have added the 7 days since this the comparison point.

5. The authors may need to include measures taken to verify and or validate the dates in the outcomes sub-section of the methods and data section of the manuscript even if appendix 1 contains all sources per country.

**Response**

Thank you. We have added as a second source to verify our compilation, the open-source depository Worldometer.

6. The authors should provide information on the rationale for compiling and analyzing data on 172 countries only. Does it mean that these 172 countries were the only countries who implemented national decision of school closure before 1st of May 2020.

**Response**

We tracked all 194 state members of United Nations’ from January 1^st^, 2020 to June 1^st^, 2022. In that period date we detected information for 186 countries, which correspond to 99,65% of the total world population. We added this information to be more explicit about how large the sample of the study is.
